# Supplementary material for: Prevalence and associated factors of microbial water quality from drinking water in Ethiopia: A systematic review and meta-analysis protocol
Source: PLoS One. 2025 Mar 4;20(3):e0313650. doi: 10.1371/journal.pone.0313650 (PMC11878905; doi:10.1371/journal.pone.0313650)
Supplement: Attachment — Submitted filename: Review PLOS ONE 170524.docx [file pone.0313650.s002.docx]

**Prevalence and associated factors of microbial water quality from drinking water in**

**Ethiopia: A Systematic Review and Meta-Analysis Protocol**

This study protocol examined the overall prevalence and predictors of fecal contamination in drinking water in Ethiopia. However, certain aspects of the materials and methods, including the study settings, sample sizes, and standard methods used for bacterial isolation, need to be clarified before publication.

Major criticism

- Was this study registered in PROSPERO?
- The term "household level of water" should be clarified.
- What automation tools were used in this study?
- The number and expertise of reviewers involved in article selection should be specified.
- How many reviewers are used for article selection? This point should be clarified.
- The rationale for selecting only 10% of the articles to assess data accuracy needs clarification.
- In Table 2, why will the language be included, since this study focuses only on the English language?
- The differences between study design, study setting, and study type need to be clearly defined.
- The factors/stratum used for the subgroup analysis should be identified.
- The term “microbial water quality” is broad; this study only reports the prevalence of fecal coliforms.
- The sources of drinking water should be specified, noting that water treatment affects bacterial contamination reduction.
- Standards/guidelines for fecal coliform contamination in drinking water should be included.

Minor criticism

- Change "Drinking water Availability" to "Drinking water availability".
- Ensure consistency in font usage.
- Italicize the names of bacterial species: Escherichia, Klebsiella, Enterobacter, and Citrobacter.
- Correct "Odd ratio" to "odds ratio".
- Change "Study time" to "duration of study".
- Specify the full name of the GBI quality parameter.
- Use "fecal coliforms" in plural form.
