# Supplementary material for: Prevalence and associated factors of microbial water quality from drinking water in Ethiopia: A systematic review and meta-analysis protocol
Source: PLoS One. 2025 Mar 4;20(3):e0313650. doi: 10.1371/journal.pone.0313650 (PMC11878905; doi:10.1371/journal.pone.0313650)
Supplement: Attachment — Submitted filename: Response to Reviewers.docx [file pone.0313650.s004.docx]

**Dear editor and reviewers,**

Response: The authors are thankful for the detailed and specific comments on the protocol. As it is a protocol, we strongly believe that this will shape our direction for the systematic review to be conducted. Most comments focused on the introduction, methodology and discussion section as well as PLOS ONE's style of writing or requirements for publication. Therefore, detailed and extensive revision is made regarding introduction, methodology and discussion section as well as PLOS ONE's style requirements formatting, inclusion criteria, PRISMA flow diagram, heterogeneity, removing overlapping texts or duplicates, expand the introduction and discussion, supporting information, data availability and others. With all this revision, we believe that the protocol will enable us conduct a standard systematic review of scientific interest. Specific responses and revisions made are provided below and highlighted in the main document. Hopefully, the editor will consider the revised protocol for further review process.

**Prevalence and associated factors of microbial water quality from drinking water in Ethiopia: A Systematic Review and Meta-Analysis Protocol**

To: editor,

Above all, we thank you for your constructive suggestions and comments on this manuscript that would improve the substance and content of the protocol. We considered each comment and clarification question of editor on the manuscript thoroughly. Our point-by-point responses for each comment and question are described in detail on the following pages. All newly modified changes were highlighted within the main manuscript. Please see the red font colour in the clean revised manuscript.

Editor Comments and Suggestions

1. When submitting your revision, we need you to address these additional requirements.

Please ensure that your manuscript meets PLOS ONE's style requirements, including those for file naming. The PLOS ONE style templates can be found at here.

Response: Thanks a lot for your eagle eyes! The editor is correct. We are very grateful for this implicit comment and suggestion. Thank you so much for such valuable intellectual input. Now, based on your suggestions and comments, we have thoroughly revised and edited all parts of our manuscript depending on PLOS ONE's style requirements. The newly modified change was highlighted with a red font color in the cleanly revised manuscript.

2. We noticed you have some minor occurrence of overlapping text with the following previous publication(s), which needs to be addressed. In your revision ensure you cite all your sources (including your own works), and quote or rephrase any duplicated text outside the methods section. Further consideration is dependent on these concerns being addressed.

Response: Thanks a lot for your eagle eyes! The editor is correct. We are very grateful for this implicit comment and suggestion. Thank you so much for such valuable intellectual input. Now, based on your suggestions and comments, we have thoroughly, cited, quoted, revised, paraphrased and edited all parts of our manuscript. The newly modified change was highlighted with a red font color in the cleanly revised manuscript.

3. When completing the data availability statement of the submission form, you indicated that you will make your data available on acceptance. We strongly recommend all authors decide on a data sharing plan before acceptance, as the process can be lengthy and hold up publication timelines. Please note that, though access restrictions are acceptable now, your entire data will need to be made freely accessible if your manuscript is accepted for publication. This policy applies to all data except where public deposition would breach compliance with the protocol approved by your research ethics board. If you are unable to adhere to our open data policy, please kindly revise your statement to explain your reasoning and we will seek the editor's input on an exemption. Please be assured that, once you have provided your new statement, the assessment of your exemption will not hold up the peer review process.

Response: Thank you very much. The editor is perfect. We are grateful for your robust and rigorous comments and suggestions. Hence, the authors utterly share the concerns and comments of editor. Thus, now with great appreciation, the authors considered your valid concern and explicitly incorporation of data availability statement of the submission form during submission. The newly modified change was highlighted with a red font color in the cleanly revised manuscript

4. Please include captions for your Supporting Information files at the end of your manuscript, and update any in-text citations to match accordingly. Please see our Supporting Information guidelines for more information

Response: Thank you very much. The editor is perfect. We are grateful for your robust and rigorous comments and suggestions. Hence, the authors absolutely share the concerns and comments of editor. Thus, now with great appreciation, the authors considered your valid concern and explicitly incorporation of Supporting Information files at the end of manuscript with perfect captions and in-text citations. The newly modified change was highlighted with a red font color in the cleanly revised manuscript.

5. **Additional Editor Comments**:

Please check comments from Reviewers and update your draft accordingly. Even though you state that this study protocol is for Ethiopia, but I think that the protocol should be sufficient to be implemented in different context/countries. So, you may cite other drinking water quality study in other regions outside Ethiopia to make this protocol and your introduction broader. In short, expand a bit the introduction and discussion of your study protocol to broader context outside Ethiopia.

Response: Thanks a lot for your eagle eyes! The editor is correct. We are very grateful for this implicit comment and suggestion. Thank you so much for such valuable intellectual input. Now, based on your suggestions and comments, we have thoroughly expanded and broadened introduction and discussion section of the manuscripts by cited and included different drinking water quality study that conducted outside of Ethiopia, as well as revised and edited all parts of our manuscript. The newly modified change was highlighted with a red font color in the cleanly revised manuscript.

**To Reviewers’**

Above all, we thank you for your constructive comments and helpful suggestions that helped us to improve and enrich our protocol. Here in the below, we have pointed out how authors incorporated your valuable comments, suggestions and concerns one by one. Thank you so much.

Reviewer #1 General comment

1. Prevalence and associated factors of microbial water quality from drinking water in Ethiopia: A Systematic Review and Meta-Analysis Protocol
This study protocol examined the overall prevalence and predictors of fecal contamination in drinking water in Ethiopia. However, certain aspects of the materials and methods, including the study settings, sample sizes, and standard methods used for bacterial isolation, need to be clarified before publication.

Response: thank you very much; we believe that the study focused on fecal coliform contamination of drinking water in Ethiopia. Fecal coliforms are purely bacteria.

2. Was this study registered in PROSPERO?

Response: Sure, it is registered in PROSPERO CRD42024537804.

3. The term "household level of water" should be clarified.

Response: Definitely! The reviewer is correct. We are grateful for this implicit comment and suggestion. The authors critically reviewed this vital comment and corrected the necessary modification as per your robust and critical comments. The newly modified change was highlighted with red font color in the clean revised main manuscript

4. What automation tools were used in this study?

Response: Well thought comments and suggestions! The reviewer is perfect. The authors critically reviewed this vital comment and corrected the necessary modification as per your valuable and critical comments. The automation tool was reference manager software (EndNote X9).The newly modified change was highlighted with red font color in the clean revised main manuscript/PRISMA flow diagram.

5. The number and expertise of reviewers involved in article selection should be specified. How many reviewers are used for article selection? This point should be clarified.

Response: Thanks a lot for your eagle eyes!  The reviewer is correct. We are very grateful for this implicit comment and suggestion. Thank you so much for such a valuable intellectual input. The newly modified change was highlighted with red font color in the clean revised main manuscript that incorporated two independent reviewers (ADW and BMA) were involved for article selection. Any discrepancy was resolved by discussion between two reviewers.

6. The rationale for selecting only 10% of the articles to assess data accuracy needs clarification.

Response: Thank you very much for your question. We are grateful for this implicit comment. To assess the correctness of the extracted data, take 10 % of the included study from each reviewer separately, due to the fact that complete texts would undergo the same review procedure. This is scientifically sound and used by different scholars.

7. In Table 1, why will the language be included, since this study focuses only on the English language?

Response: Thank you so much to pointing out this. The reviewer is perfect. We are very grateful for this implicit comment and suggestion. Thank you so much for such a valuable intellectual input. Authors critically considered this valid input. Now, the newly modified change was highlighted with red font color in the clean revised manuscript.

**Data extraction table for systematic review and meta-analysis study**

| Author | Publication Year | Publication Source | Region | Study design | Study setting | Study duration | Sample size | No of samples tested +( FC) | GBI quality score | Prevalence (%),( CI) | Associated factors  OR (CI), or 2X2 classification of predictors. |
| --- | --- | --- | --- | --- | --- | --- | --- | --- | --- | --- | --- |
|  |  |  |  |  |  |  |  |  |  |  |  |
|  |  |  |  |  |  |  |  |  |  |  |  |
|  |  |  |  |  |  |  |  |  |  |  |  |

Table1: data extraction table for Characteristics of the studies included in the systemic review and meta-analysis

8. The differences between study design, study setting, and study type need to be clearly defined.

Response: Definitely! Many thanks for it. We are grateful for this crucial remark. Reviewer, you are flawless. This significant observation of highlights was thoroughly addressed in the updated paper. The newly modified change was highlighted with red font color in the clean revised manuscript.

9. The factors/stratum used for the subgroup analysis should be identified.

Response: Thank you so much for such valuable intellectual input. The reviewer is perfect. We are very grateful for this implicit comment and suggestion. Thank you so much for such valuable intellectual input. The factors that used for subgroup analysis was to detect the source of heterogeneity in the outcome variables. We used subgroup analysis and Meta regression if possible depending on the data types those is categorical type in this review protocol. The authors critically considered this valid input. The newly modified change was highlighted with a red font color in the cleanly revised manuscript.

10. The term “microbial water quality” is broad; this study only reports the prevalence of fecal coliforms.

Response: Many thanks for it, since fecal coliforms are the large subset and parts of the microbial colony that need to be tested in drinking water quality. The authors used the term “microbial water quality in this systematic review and meta-analysis study.

11. The sources of drinking water should be specified, noting that water treatment affects bacterial contamination reduction.

Response: Thanks a lot for your eagle eyes!  The reviewer is correct. We are very grateful for this implicit comment and suggestion. Thank you so much for such a valuable intellectual input. The authors critically considered this valid input. The newly modified change was highlighted with a red font color in the cleanly revised manuscript.

12. Standards/guidelines for fecal coliform contamination in drinking water should be included.

Response: Thank you so much for pointing this out. The reviewer is flawless. We greatly appreciate your implied observation and recommendation. The authors include standards for fecal coliforms contamination in drinking water. WHO guidelines/Standards and Ethiopian standard agency for fecal coliforms contamination of drinking water were must not be detectable in any 100 ml water sample. The newly modified change was highlighted with red font color in the clean revised manuscript.

13. Change "Drinking water Availability" to "Drinking water availability".

Response: Thanks a lot for your eagle eyes!  The reviewer is correct. We are very grateful for this implicit comment and suggestion. Thank you so much for such a valuable intellectual input. The authors critically considered this valid input. The newly modified change was highlighted with a red font color in the cleanly revised manuscript.

14. Ensure consistency in font usage.

Response: Thank you, a lot, for pointing out this!  I am really grateful for your insightful comments. The author fully offers the utterly shares the comments and suggestion, which significantly improve the manuscript's quality. We made the font size of the text consistent throughout the whole manuscript. The newly modified change was highlighted with red font color in the clean revised manuscript.

15. Italicize the names of bacterial species: Escherichia, Klebsiella, Enterobacter, and Citrobacter, and Correct "Odd ratio" to "odds ratio".

Response: Thank you, a lot, for pointing out this!  Thank you so much for such valuable intellectual input. The Author utterly shares the comments and suggestion which immensely enhance the manuscript quality. The newly modified change was highlighted with red font color in the clean revised manuscript.

16. Change "Study time" to "duration of study".

Response: Definitely! The reviewer is correct. We are grateful for this implicit comment and suggestion. The authors critically reviewed the section. The newly modified change was highlighted with red font color in the clean revised manuscript

17. Specify the full name of the GBI quality parameter.

Response: Absolutely! We really appreciated this important observation. We are at loss for describing your commitments and dedication. The Authors utterly shares the comments and suggestion which immensely enhance the manuscript, subsequently the abbreviations were not present in abstract section. The newly modified change was highlighted with red font color in the clean revised manuscript.

18. Use "fecal coliforms" in plural form.

Response: Thank you very much. The reviewer is perfect. We are grateful for your robust and rigorous comments and suggestions. Hence, the author utterly shares the concerns and comments of the reviewer. Thus, now, with great appreciation, the authors have considered your valid concern and explicitly incorporated your input into the clean revised manuscript, highlighted with a red font color.

**Reviewer #2:**

This study protocol, entitled systematic review and meta-analysis on the prevalence and contributing factors of fecal coliform contamination in drinking water in Ethiopia addresses a critical public health issue. The methodology that was outlined is comprehensive and follows established guidelines, demonstrating a thorough approach to this important topic. However, I have identified several areas where the manuscript could be strengthened to enhance its clarity, depth, and overall impact. Please find below my detailed comments and suggestions for improvement.

19. The search terms should contain the species of fecal coliform bacteria, including Escherichia, Klebsiella, Enterobacter, and Citrobacter. This can integrate all possible publications conducted in Ethiopia relevant to the exact microbial quality of drinking water in this country.

Response: Thank you very much. The objective of this study mainly focused on the prevalence of fecal coliforms contamination from drinking water in Ethiopia in cumulative way (second stage of test) rather than including each species study, to show unsanitary condition and fecal contamination of drinking water, since it is a broad indicator. During water quality test there are three stage, identified total coliforms, fecal coliforms, and E.coli. In the first and second stage the lab professionals, identified total coliforms, and fecal coliforms, next the third stage from fecal coliforms the lab professionals incubate further at 44.5°C to find the species it may be E.coli, Klebsiella, Enterobacter, Citrobacter or any other viable bacteria or microorganisms. So this review protocol mainly focused on fecal coliform studies that conducted at second stage of detection, but not included studies that on specific species, to know the overall burden of fecal coliforms contaminations of drinking water in Ethiopia, as a result it is a much broad proxy indicators for many organisms.

**Search terms**

To increase the probability of all-inclusiveness of the findings, search terms were developed and combined using Boolean operators as follows:

The search terms used were “prevalence," “associated factors," “microbial water quality," “bacterial water quality," and “drinking water in Ethiopia. Prevalence and associated factors of microbial water quality or associated factors of bacterial water quality from drinking water in Ethiopia. We would undertake advanced searching by combining the search terms using Boolean operators.

**Search detail**

((("epidemiology"[MeSH Subheading] OR "epidemiology"[All Fields] OR "prevalence"[All Fields] OR "prevalence"[MeSH Terms] OR "prevalance"[All Fields] OR "prevalences"[All Fields] OR "prevalence s"[All Fields] OR "prevalent"[All Fields] OR "prevalently"[All Fields] OR "prevalents"[All Fields]) AND ("associate"[All Fields] OR "associated"[All Fields] OR "associates"[All Fields] OR "associating"[All Fields] OR "association"[MeSH Terms] OR "association"[All Fields] OR "associations"[All Fields]) AND ("factor"[All Fields] OR "factor s"[All Fields] OR "factors"[All Fields]) AND ("microbial"[All Fields] OR "microbially"[All Fields] OR "microbials"[All Fields]) AND ("water quality"[MeSH Terms] OR ("water"[All Fields] AND "quality"[All Fields]) OR "water quality"[All Fields])) OR (("associate"[All Fields] OR "associated"[All Fields] OR "associates"[All Fields] OR "associating"[All Fields] OR "association"[MeSH Terms] OR "association"[All Fields] OR "associations"[All Fields]) AND ("factor"[All Fields] OR "factor s"[All Fields] OR "factors"[All Fields]) AND ("bacterial"[All Fields] OR "bacterially"[All Fields] OR "bacterials"[All Fields]) AND ("water quality"[MeSH Terms] OR ("water"[All Fields] AND "quality"[All Fields]) OR "water quality"[All Fields]) AND ("drinking water"[MeSH Terms] OR ("drinking"[All Fields] AND "water"[All Fields]) OR "drinking water"[All Fields]) AND ("Ethiopia"[MeSH Terms] OR "Ethiopia"[All Fields] OR "Ethiopia s"[All Fields]))) AND ("2000/01/01"[PubDate]: "2024/03/11"[PubDate]).

All searches will be restricted to the years “between” 2000 and 2024 and only in the English language; however, each online database varies in terms of the earliest articles available online.

Grey literature Search terms will be modified in the databases where required. Basic terms will be used for the grey literature. Bibliographies of included studies and relevant reviews will be searched. Based on the resources available, the titles and abstracts from selected journals will be reviewed. These papers will be selected based on the number of eligible articles identified in each paper in prior searches.

20. In addition, in PRISMA flow chart, I recommend that publications that were not published in English be excluded at the first step of the screening process.

Response: Thank you so much for pointing this out! Many thanks for it. We are grateful for this crucial remark. Reviewer, you are flawless. This significant observation was thoroughly addressed in the updated paper. Please refer to the neatly corrected content and the updated red font color.

**Study selection flow diagram**

Identification of studies via other methods

**Identification of studies via databases**

**Identification**

Records identified from: Databases (n =)

PubMed (n=), Scopus (n=), Google scholar (n=), web of science (n=), science direct.(n=)

Records screened

(n =)

Reports sought for retrieval

(n =)

**Screening**

Identified from:

Websites/Google (n =)

Reference list (n =)

Records removed *before screening*: Duplicate records removed

By manual (n = )

By Software (EndNote X9) (n =)

Records excluded (n =)

Based on title and abstracts (n=)

Reports sought for retrieval

(n =	)

Reports not retrieved based on full text

(n =)

Reports assessed for eligibility

(n =)

Reports excluded:

Study area (n =)

Study period (n =)

Study design (n =)

Ineligibility of the outcomes (n=)

Report assess for eligibility

(n =)

Studies included in systematic review and meta-analysis (n =)

**Included**

Figure1. Study selection flow diagram for this systematic review and meta-analysis

Sources: doi: 10.1136/bmj.n71.

21. Lastly, the criteria that will be used for subgroup analysis does not indicated in the protocol.

Response:

Definitely! The reviewer is correct. We are grateful for this implicit comment and suggestion. The authors critically reviewed this vital comment and corrected the missing data modification as per your robust and critical comments. To know the source of heterogeneity in the outcome variable, we used subgroup analysis and meta-regression depending on the type of data that are categorical data including the following characteristics, region (Amhara, Oromia, Tigrai…), study setting (urban vs rural), study design (cross sectional, case control and cohort), source of publication (published and unpublished), and publication year (before 2015 and equal or more than 2015 until 2024) etc..). The newly modified change was highlighted with red font color in the clean revised main manuscript.

End of the response

Thank you in advance!!!
