# Supplementary material for: Prevalence and associated factors of microbial water quality from drinking water in Ethiopia: A systematic review and meta-analysis protocol
Source: PLoS One. 2025 Mar 4;20(3):e0313650. doi: 10.1371/journal.pone.0313650 (PMC11878905; doi:10.1371/journal.pone.0313650)
Supplement: Attachment — Submitted filename: Response_to_Reviewers_auresp_2.docx [file pone.0313650.s005.docx]

**Dear editor and reviewers,**

Response: The authors are thankful for the detailed and specific comments on the protocol. As it is a protocol, we strongly believe that this will shape our direction for the systematic review to be conducted. Most comments focused on the methodology and figure submission formatting style of PLOS ONE' for publication. Therefore, detailed and extensive revision is made regarding methodology and figure submission formatting, and corresponding author contacting time. With all this revision, we believe that the protocol will enable us conduct a standard systematic review of scientific interest. Specific responses and revisions made are provided below and highlighted in the main document. Hopefully, the editor will consider the revised protocol for publication.

**Prevalence and associated factors of microbial water quality from drinking water in Ethiopia: A Systematic Review and Meta-Analysis Protocol**

To: editor,

Above all, we thank you for your constructive suggestions and comments on this manuscript that would improve the substance and content of the protocol. We considered each comment and clarification question of editor on the manuscript thoroughly. Our point-by-point responses for each comment and question are described in detail on the following pages. All newly modified changes were highlighted within the main manuscript. Please see the red font colour in the clean revised manuscript.

Editor Comments and Suggestions

1. If you would like to make changes to your financial disclosure, please include your updated statement in your cover letter.

Response: Thanks a lot for your suggestions! This revised manuscript has no any conflict of interest exists.

2. Guidelines for resubmitting your figure files are available below the reviewer comments at the end of this letter. While revising your submission, please upload your figure files to the Preflight Analysis and Conversion Engine (PACE) digital diagnostic tool

Response: Thanks a lot for your eagle eyes! The editor is correct. We are very grateful for this implicit comment and suggestion. Thank you so much for such valuable intellectual input. Now, based on your suggestions and comments, we have thoroughly, uploaded in to the Preflight Analysis and Conversion Engine (PACE) digital diagnostic tool to change the figure based on the PLOS ONE submission guideline. The newly modified change was highlighted with a red font color in the cleanly revised manuscript.

3. If applicable, we recommend that you deposit your laboratory protocols in [protocols.io](http://protocols.io/) to enhance the reproducibility of your results. Protocols.io assigns your protocol its own identifier (DOI) so that it can be cited independently in the future. Additionally, PLOS ONE offers an option for publishing peer-reviewed Lab Protocol articles, which describe protocols hosted on [protocols.io](http://protocols.io/).

Response: Thank you very much. The editor is perfect, but this protocol is not laboratory protocol.

4. Please review your reference list to ensure that it is complete and correct. If you have cited papers that have been retracted, please include the rationale for doing so in the manuscript text, or remove these references and replace them with relevant current references. Any changes to the reference list should be mentioned in the rebuttal letter that accompanies your revised manuscript. If you need to cite a retracted article, indicate the article’s retracted status in the References list and also include a citation and full reference for the retraction notice.

Response: Thank you very much. The editor is perfect. We are grateful for your robust and rigorous comments and suggestions. The reference list is complete and correct. No need of any change in reference list.

**To Reviewers’**

Above all, we thank you for your constructive comments and helpful suggestions that helped us to improve and enrich our protocol. Here in the below, we have pointed out how authors incorporated your valuable comments, suggestions and concerns one by one. Thank you so much.

Reviewer #1

1. The questions and concerns I addressed are thoroughly explained in the manuscript, making it ready for publication.

Response: Thank you very much for your implicit comment and suggestion for this systematic review and meta-analysis protocol finalization. Your effort is much appreciated.

Reviewer #2

1. Your manuscript is well prepared and relevant to objectives. I appreciate to your corrections. Here is a list of some suggestions.

Response: Thank you very much for your crucial remark, comment and suggestion for this systematic review and meta-analysis protocol finalization. Your effort is much respected.

2. I recommend that you review grey literature authored by at least two individuals, given the generally lower quality of these studies.

Response: Definitely! The reviewer is correct. We are grateful for this implicit comment and suggestion. The authors critically reviewed this vital comment and corrected the necessary modification as per your robust and critical comments. The newly modified change was highlighted with red font color in the clean revised main manuscript

3. When reaching out to corresponding authors for missing information, I suggest making up to three contact attempts to maximize your chances of receiving a response

Response: Well thought comments and suggestions! The reviewer is flawless. The authors critically reviewed this vital comment and corrected the necessary modification as per your valuable and critical comments. We will try up to three attempts to reach out the corresponding author to maximize the response. The newly modified change was highlighted with red font color in the clean revised main manuscript.

4. To enhance your search results, consider adding additional search terms related to "P" in the PICO statement, such as "occurrence," "detection," "proportion," or "rate," to capture a broader range of research.

Response: Thanks a lot for your eagle eyes!  The reviewer is correct. We are very grateful for this implicit comment and suggestion. Thank you so much for such a valuable intellectual input..

**Thank you very much for reviewing our work!!!**
