# Supplementary material for: Prevalence and associated factors of microbial water quality from drinking water in Ethiopia: A systematic review and meta-analysis protocol
Source: PLoS One. 2025 Mar 4;20(3):e0313650. doi: 10.1371/journal.pone.0313650 (PMC11878905; doi:10.1371/journal.pone.0313650)
Supplement: Attachment — Submitted filename: Response_to_Reviewers_auresp_3.docx [file pone.0313650.s006.docx]

**Dear editor and reviewer,**

Response: The authors are thankful for the detailed and specific comments on the protocol. As it is a protocol, we strongly believe that this will shape our direction for the systematic review to be conducted. Most comments focused on the introduction section and study selection parts for publication. Therefore, detailed and extensive revision is made regarding introduction and study selection. With all this revision, we believe that the protocol will enable us conduct a standard systematic review of scientific interest. Specific responses and revisions made are provided below and highlighted in the main document. Hopefully, the editor will consider the revised protocol for publication.

**Prevalence and associated factors of microbial water quality from drinking water in Ethiopia: A Systematic Review and Meta-Analysis Protocol**

To: editor,

Above all, we thank you for your constructive suggestions and comments on this manuscript that would improve the substance and content of the protocol. We considered each journal requirements and suggestion of editor on the manuscript thoroughly. Our point-by-point responses for each journal requirements and suggestion are described in detail on the following pages.

Editor Comments and Suggestions

1. If you would like to make changes to your financial disclosure, please include your updated statement in your cover letter.

Response: Thanks a lot for your suggestions! This revised manuscript has no any conflict of interest exists and have no any change made from the previous one.

2. If applicable, we recommend that you deposit your laboratory protocols in [protocols.io](http://protocols.io/) to enhance the reproducibility of your results. Protocols.io assigns your protocol its own identifier (DOI) so that it can be cited independently in the future. Additionally, PLOS ONE offers an option for publishing peer-reviewed Lab Protocol articles, which describe protocols hosted on [protocols.io](http://protocols.io/).

Response: Thank you very much. The editor is perfect, but this protocol is not laboratory protocol.

3. Please review your reference list to ensure that it is complete and correct. If you have cited papers that have been retracted, please include the rationale for doing so in the manuscript text, or remove these references and replace them with relevant current references. Any changes to the reference list should be mentioned in the rebuttal letter that accompanies your revised manuscript. If you need to cite a retracted article, indicate the article’s retracted status in the References list and also include a citation and full reference for the retraction notice.

Response: Thank you very much. The editor is perfect. We are grateful for your robust and rigorous comments and suggestions. The reference list is complete and correct. No need of any change in reference list.

**To Reviewer**

Above all, we thank you for your constructive comments and helpful suggestions that helped us to improve and enrich our protocol. Here in the below, we have pointed out how authors incorporated your valuable comments, suggestions and concerns one by one. Thank you so much.

Reviewer #2

1. Your manuscript is well-organized and aligns with the stated objectives. Below are some additional suggestions.

Response: Thank you very much for your crucial remark, comment and suggestion for this systematic review and meta-analysis protocol finalization. Your effort is much appreciated and respected.

2.  Line 67: Please provide the full name of "MDG.".

Response: Definitely! The reviewer is correct. We are grateful for this implicit comment and suggestion. The authors critically reviewed this vital comment and corrected the necessary modification as per your robust and critical comments. The newly modified change was highlighted with red font color in the clean revised main manuscript

3. Line 70: The term "E. coli" should be written in full at its first mention

Response: Well thought comments and suggestions! The reviewer is flawless. The authors critically reviewed this vital comment and corrected the necessary modification as per your valuable and critical comments. We rewrite the word in full text. The newly modified change was highlighted with red font color in the clean revised main manuscript.

4. Line 84: Clarify whether the bacteria found in North Carolina were fecal coliforms, E. coli, or both.

Response: Thanks a lot for your eagle eyes!  The reviewer is correct. We are very grateful for this implicit comment and suggestion. Thank you so much for such a valuable intellectual input. The bacteria were fecal coliforms not E.coli and found in Nepal but not in North Carolina. We edited and revised the main manuscript. The newly modified change was highlighted with red font color in the clean revised main manuscript.

5. Line 85: "Escherichia coli" should be abbreviated and italicized, as you introduced it earlier in line 70.

Response: Thank you, a lot, for pointing out this!  I am really grateful for your insightful comments. The authors fully offers the utterly shares the comments and suggestion, which significantly improve the manuscript's quality. We made abbreviated and italicized the word "Escherichia coli". The newly modified change was highlighted with red font color in the clean revised manuscript.

6. Lines 89 and 94: Since "TTC" is previously defined in lines 77-78, use the abbreviation throughout.

Response: Thank you, a lot, for pointing out this!  Thank you so much for such valuable intellectual input. The Authors utterly shares the comments and suggestion which immensely enhance the manuscript quality. The newly modified change was highlighted with red font color in the clean revised manuscript.

7.  Line 92: The word "and" should not be italicized.

Response: Definitely! The reviewer is correct. We are grateful for this implicit comment and suggestion. The authors were edited and corrected the word "and" in normal writing style. The newly modified change was highlighted with red font color in the clean revised manuscript

8. Line 103: The term "Scooping" should be written in lowercase.

Response: Definitely! The reviewer is correct. We are grateful for this implicit comment and suggestion. The authors were edited and corrected the word "Scooping". The newly modified change was highlighted with red font color in the clean revised manuscript

9. Lines 196 and 222: Consider adding an additional reviewer to resolve any discrepancies in the final decision. Lines 280-281: If the authors choose to use only two reviewers for resolving discrepancies, this should be noted as a potential limitation.

Response: Absolutely! We really appreciate this important observation. We are at loss for describing your commitments and dedication. The Authors utterly shares the comments and suggestion which immensely enhance the manuscript, subsequently use of only two reviewers were mentioned in the limitation parts of the manuscript. The newly modified change was highlighted with red font color in the clean revised manuscript.

**Thank you very much for reviewing our work!!!**
